# Modeling Tuberculosis Dynamics, Detection and Control in Cattle Herds

**DOI:** 10.1371/journal.pone.0108584

**Published:** 2014-09-25

**Authors:** Mohammed El Amine Bekara, Aurélie Courcoul, Jean-Jacques Bénet, Benoit Durand

**Affiliations:** 1 University Paris Est, Anses, Laboratory of Animal Health, Epidemiology Unit, Maisons-Alfort, France; 2 University Paris Est, National Veterinary School of Alfort (ENVA), EpiMAI Unit, Maisons-Alfort, France; Imperial College London, United Kingdom

## Abstract

Epidemiological models are key tools for designing and evaluating detection and control strategies against animal infectious diseases. In France, after decades of decrease of bovine tuberculosis (bTB) incidence, the disease keeps circulating. Increasing prevalence levels are observed in several areas, where the detection and control strategy could be adapted. The objective of this work was to design and calibrate a model of the within-herd transmission of bTB. The proposed model is a stochastic model operating in discrete-time. Three health states were distinguished: susceptible, latent and infected. Dairy and beef herd dynamics and bTB detection and control programs were explicitly represented. Approximate Bayesian computation was used to estimate three model parameters from field data: the transmission parameter when animals are inside (β_inside_) and outside (β_outside_) buildings, and the duration of the latent phase. An independent dataset was used for model validation. The estimated median was 0.43 [0.16–0.84] month^−1^ for β_inside_ and 0.08 [0.01–0.32] month^−1^ for β_outside_. The median duration of the latent period was estimated 3.5 [2]–[8] months. The sensitivity analysis showed only minor influences of fixed parameter values on these posterior estimates. Validation based on an independent dataset showed that in more than 80% of herds, the observed proportion of animals with detected lesions was between the 2.5% and 97.5% percentiles of the simulated distribution. In the absence of control program and once bTB has become enzootic within a herd, the median effective reproductive ratio was estimated to be 2.2 in beef herds and 1.7 in dairy herds. These low estimates are consistent with field observations of a low prevalence level in French bTB-infected herds.

## Introduction

Bovine tuberculosis (bTB) is a chronic animal disease most often caused by *Mycobacterium bovis*, that mainly affects the respiratory system [1]. The main route of transmission of the infection between cattle is the respiratory route [2]. In France, a control program for bTB became mandatory in 1965. Detection of infected herds was based on an annual screening of animals using skin tests, and on routine inspection of carcasses at slaughter for bTB-like lesions (with subsequent isolation of *M. bovis* at the laboratory). Since 1990, the control program was reinforced by the compulsory screening (using skin tests) of animals introduced in bTB-free herds, these animals always originating from other bTB-free herds. In 1999, herd prevalence fell below 0.1% and the slaughter of all cattle in infected herds (termed below “total slaughter”) was introduced in the control program. In 2001, France was declared officially free of bTB by the European Union. However, in recent years, an increase in bTB incidence has been observed in some departments. Similar trends have been observed in other European countries. In Great Britain and Ireland, the control programs became mandatory in 1950 and 1957, respectively. They were also based on an annual screening by skin test, the slaughter of positive animals and the inspection of carcasses at the slaughterhouse [3]. bTB incidence decreased in both countries, but the epidemiological situation began to deteriorate gradually from the 1980s, partly because of the existence of a wildlife reservoir in badger populations [4]. In France, such a wildlife reservoir has not been identified, although wild boars, red deer and badgers with *M. bovis* lesions have been found in several departments. Unlike France, Great Britain or Ireland, there are countries where “test and slaughter” control programs were successful in eradicating bTB: Sweden was considered bTB-free in 1958, after 40 years of this type of control program [5] (sporadic cases have been reported afterwards, the last one in 1978 [6]).

In France, data collected over 35 years, between 1965 and 2000, have shown parallel evolutions of herd management practices (with a disappearance of family farms and a professionalization of breeders) and of herd structures (with changes in herd types with a switch from dairy to beef and increasing herd sizes). Besides the effectiveness of control programs, the decrease of bTB incidence between 1965 and 2000 could be partly attributable to these changes in herd management practices and in herd structures [7].

Neither ante-mortem tests [8] nor post-mortem tests [6], [9] used in bTB control programs have perfect sensitivity and specificity. With the decrease of infection prevalence and because of the increase of herd sizes (multiplied on average by 3.5 between 1965 and 2000 in France [7]), ante-mortem false-positive reactions became a major problem for the surveillance and control program and its acceptability by breeders. This led to an increasing complexity of testing procedures over the past fifteen years, with combinations of several tests: single intradermal tuberculin test (SITT), single intradermal comparative cervical tuberculin test (SICCT) and gamma interferon test (γIFN). Additionally, total slaughter has been replaced in some areas by the slaughter of reactive animals only. All these adaptations allowed reducing the number of animals slaughtered due to false-positive tests, the counterpart being the burdensomeness of control programs due to prolonged restriction of cattle movements.

The failure of the eradication of bTB in France and other European countries seems therefore the result of a complex interaction between the evolution of herd management practices and herd structures, the evolution of control programs, and the implication of wildlife as a reservoir for infection in cattle.

Mathematical modelling is used for the study of complex phenomena such as the dynamics of infectious agents [10]. Diseases with a long incubation period, such as bTB, are difficult to study experimentally or in the field because of prolonged waiting times for obtaining results and the high costs of conducting experimental studies [2]. Mathematical simulation models offer the possibility to test a range of control programs in a short time and to identify the most effective one [2].

A number of mathematical models have been developed to represent the spread of bTB and assess the effectiveness of control measures, especially for wildlife (badgers, opossums and white-tailed deer) [11]. Several models have been built to simulate the dynamics of bTB within cattle herds, to quantify the importance of within-farm bTB transmission by estimating the within-herd transmission coefficient of infection [2], [12]–[14] or to evaluate detection and control strategies against bTB [2], [13], [15], [16].

Each of these models incorporates three processes that shape the within-herd bTB infection dynamics: (i) the natural history of the disease, (ii) husbandry practices and (iii) the surveillance and control program [15]. Because points (ii) and (iii) are specific to a particular context (geographical area and time period), the models mentioned above are difficult to extrapolate to other countries.

The aim of this study was to design, calibrate and validate a model of spread of *M. bovis* within a cattle herd. Parameter definition was designed to allow simulating various herd management practices as well as control programs of arbitrary complexity.

## Materials and Methods

### Ethical statement

bTB is a notifiable disease for which there are control and surveillance campaigns in France. Official methods for diagnosis of this disease are culture, PCR and histopathology. Therefore, all the datasets included in this study are issued from animals analyzed within an official context. No purpose killing of animals was performed for this study. All datasets were in complete agreement with national and European regulations. No ethical approval was necessary.

### Data

Four datasets denoted A, B, C and D were used, which had been collected at different periods (between 1980 and 2010) in 3 French departments (Nord, Dordogne, and Côte d'Or). At that time and in these departments, four specific control programs were applied, also denoted A, B, C and D (Table 1).

**Table 1:** Description of the four datasets.

Id	Data type	Number of herds (animals)		Period(geographic origin)	Control program
		Dairy	Beef		
A	Aggregated	5 (134)		1981–1983 (Nord)	Annual screening by SITT, slaughter of all cattle if>40% of SITT-positive animals, otherwise slaughter of SITT-positive only
B	Individual	1 (60)	12 (625)	2004–2006 (Dordogne)	Biennial screening by SITT confirmed by SICCT, slaughter of SICCT-positive animals, slaughter of all cattle in case of lesions and *M. bovis* isolation
	Aggregated	9 (684)	16 (1,011)		
C	Individual	3 (142)	11 (596)	2007–2010 (Dordogne)	Annual screening by SITT with second control by γIFN^1^ and confirmation by SICCT, slaughter of SICCT-positive, slaughter of all cattle in case of lesions and *M. bovis* isolation
	Aggregated	7 (530)	22 (1,344)		
D	Aggregated		29 (6,317)	2005–2009 (Côte d’Or)	Biennial screening by SICCT, slaughter of SICCT-positive animals, slaughter of all cattle in case of lesions and *M. bovis* isolation

1 To improve the specificity of the control program C, the γIFN assay used in this control program is a combination of two tests: the PPD γIFN and the ESAT-6 γIFN. The modified γIFN test can be positive (both tests are positive), negative (both tests are negative) or divergent (the results of the two tests are discordant).

The total number of herds in the four datasets was 115. All were submitted to a total slaughter. Out of all these 115 farms, detailed data for each animal (individual data) were available for 27 herds, namely the year of birth, the screening tests used, the date and results of these tests, the date of slaughter and the result of carcass inspection at the slaughterhouse (presence or absence of lesions). For the 88 remaining farms, we obtained a single data point aggregated at the herd level: the within-herd proportion of animals with detected lesions at total slaughter (Table 1). Dataset A was obtained from a case study conducted by Carnon [17]. The median percentage of animals with lesions per herd was 34.6% (min: 20.8%, max: 86.4%). Dataset B and C were obtained from the official veterinary services of Dordogne department. In dataset B, the median percentage of animals with lesions per herd was 6.4% (min: 0.1%, max 52%). In dataset C, it was 3.1% (min: 0.6%, max 43%). Data set D was obtained from the Alfort National Veterinary School. The median percentage of animals with lesions per herd was 1% (min: 0.3%, max: 5%).

### Model

**Figure 1:**
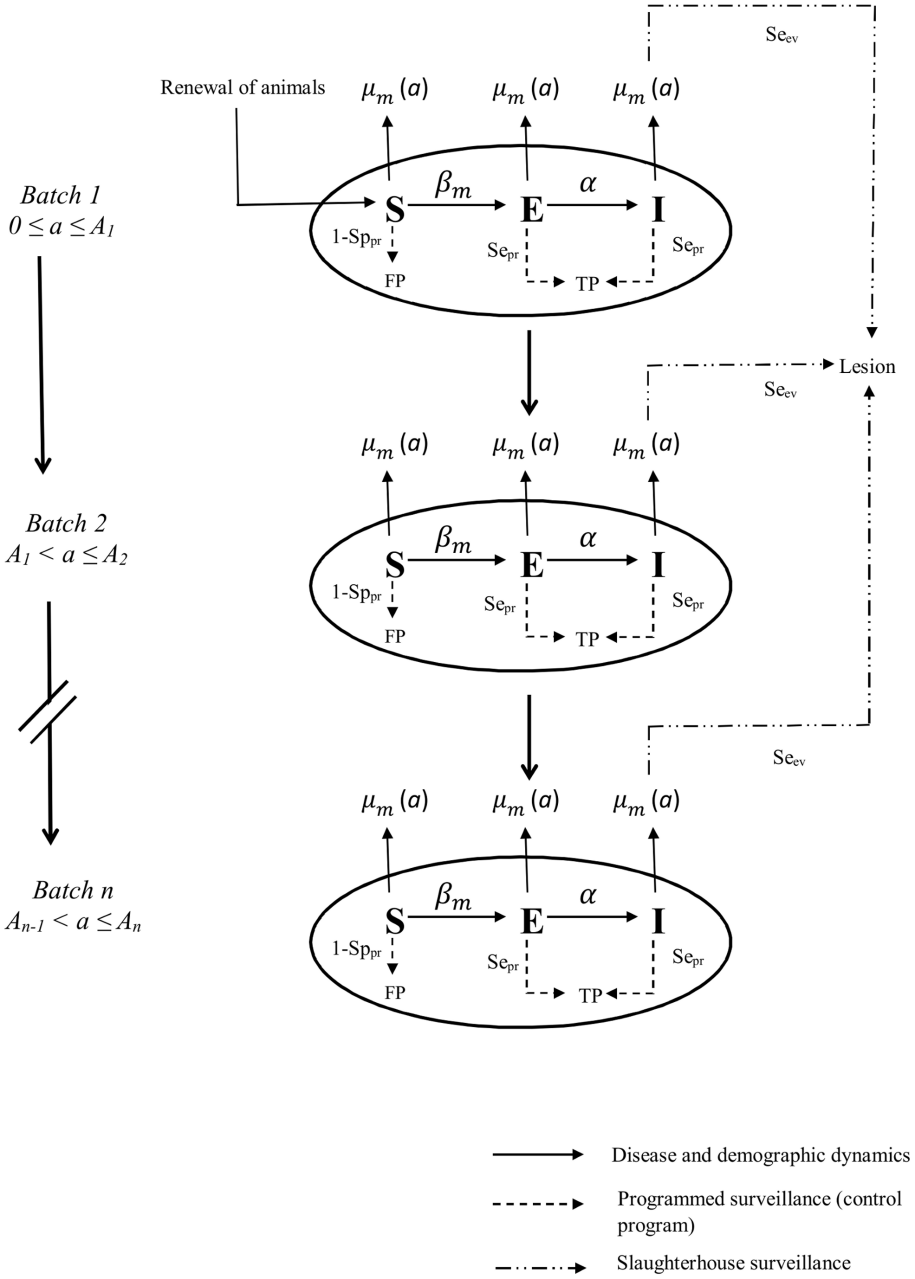
Schematic representation of the model of within-herd transmission of bTB by three processes: the demographic process (renewal of cattle, reform and change of batches), the infectious process (transitions between health states S: susceptible, E: latent, I: infectious) and the process of detection and control. a: age class in years; Batch: group of animals; m: month; µ_m_ (a): culling rate for month m and age class a; β_m_: month-specific transmission coefficient; Se_pr_: test sensitivity of step k of programmed surveillance (control program); Sp_pr_: test specificity of step k of programmed surveillance (control program); FP: false positive; TP: true positive; Se_ev_: sensitivity of passive surveillance at the slaughterhouse.

The within-herd spread of bTB was modelled using a compartmental stochastic model operating in discrete time (Figure 1). The time step was one month. Hereafter, *t* represents the current time step and *m* the current month. Each cattle was represented by its age in years i∈A = {0,…,A_max} and its health state j∈H = {S,E,I}, with: *S* (susceptible): the animal is not infected, *E* (latent): the animal is infected but does not have lesions and does not excrete bacteria, and *I* (infectious): the animal is infected, has lesions and excretes bacteria. Animals were assumed to be grouped into batches according to their age class. To each age class *i* (i∈A) was assigned a batch denoted L(*i*) (with L(i)⊂A). A given batch thus contained animals of one or several age classes. In practice, these batches are often placed by farmers on distant pastures and sometimes in separate buildings, animals from distinct batches having little contact with each other.

**Figure 2:**
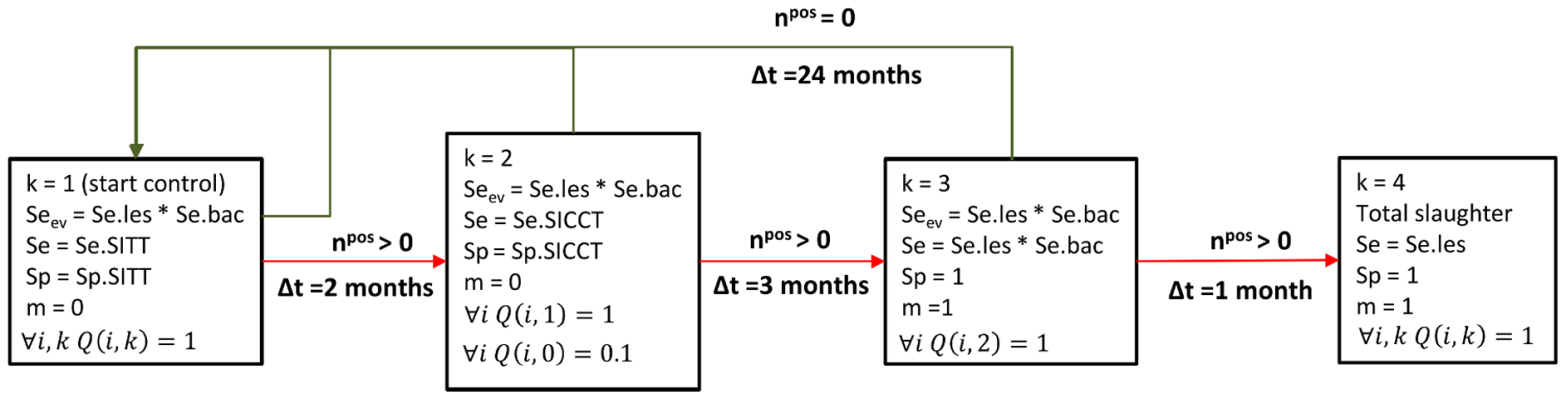
Control program B applied in the Dordogne department between 2004 and 2006. Step 1: biennial bTB screening using SITT (the herd being considered disease-free). All animals are tested (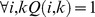). Sensitivity (Se) and specificity (Sp): those of SITT. Transition to step 2 if an animal is positive (n^pos^>0), two months later (Δt = 2 months. Step 2: confirmation of SITT positive results using SICCT. All the positive animals of step 1 are tested (µ i Q(i,1) = 1), as well as 10% of the negative animals (µ i Q(i,1) = 1). Sensitivity (Se) and specificity (Sp): those of SICCT. Transition to step 3 if an animal is positive, three months later. Step 3: slaughter of SICCT-positive animals and isolation of *M. bovis* from lesions. All the positive animals of step 2 (µ i Q(i,2) = 1) are slaughtered (m = 1) and bacterial culture is performed from observed lesions. Sensitivity (Se): sequential combination of a visual inspection at the slaughterhouse and of a bacterial culture. Transition to step 4 if an animal is positive, 1 month later. Step 4: total slaughter. All the animals (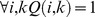) are slaughtered (m = 1). Routine detection of lesions at slaughterhouse. Sensitivity (Se_ev_): sequential combination of a visual inspection at the slaughterhouse and of a bacterial culture. If a positive animal is thus detected, regardless of the current stage of control program, transition to step 4. See table 2 for the definition of the other parameters.

The control program was represented by a succession of steps numbered from 1 to *D_max_*. During each of these steps, biological tests were performed on the entire or part of the herd; the results of these tests determined the following step. For example, in the control program B which was applied between 2004 and 2006 in the department of Dordogne (Figure 2), the first step is the yearly screening of all animals by SITT. If one animal is positive, the second step of control program is performed two months later: positive animals of step 1 are tested by SICCT, as well as a group of randomly selected negative animals. If one animal is positive in step 2, step 3 is performed 3 months later: positive animals of step 2 are slaughtered and, when lesions are observed, bacterial cultures are performed. If one animal is positive to the bacterial culture, the total slaughter (step 4) is performed 1 month later. The status of each animal with respect to the step of the control program was represented by an integer k∈D = {0,…,D_max}, with: *k* = 0 if the animal has never been tested or always had negative test results, and *k*>0 if the animal had positive results to tests performed in step *k* of the control program, and negative tests thereafter.

The state of a herd at time step *t* was represented by a triplet〈X_t_,y_t_,z_t_〉, where *y_t_* is the number of the current step of the control program (1≤ *y_t_* ≤*D_max_*), *z_t_* is the date on which the biological tests associated with this step must be performed (z*_t_*≥*t*), and *X_t_* is a state variable structured on age class, health status and step of the control program, which represents the state of the animals at time step *t*: X_t_ (i,j,k) represents the number of animals in age class *i* (i∈A), health state *j* (j∈H), which have the status *k* (k∈D) with respect to the control program.

The dynamic of infection in the herd resulted from the implementation of three sequential processes (Figure 1): the demographic process (ageing and renewal of animals), the infectious process (transmission of infection and evolution of infected animals) and the process of detection and control. A full description of the model is given in Appendix S1.

The size of the herd was assumed constant over time and the herd was assumed closed. Slaughtered animals (routine slaughter or implementation of the control program) being replaced by susceptible young animals (0–1 years old) born in the same herd. The culling rate k∈D was assumed to vary according to the month and to the age class.

We made the simplifying assumption that transmission can occur only between animals of the same batch. However, because of the ageing of animals, the composition of the batch changes every year. Animal transfers between batches then allowed the between-batch infection spread. The within-herd transmission of bTB was modelled by a frequency-dependent function. Because *M. bovis* transmission intensity was assumed to vary according to whether the animals are housed inside a stable (where the intensity of within-batch transmission is assumed to be high) or allowed to graze (where the intensity of within-batch transmission is assumed to be low), the transmission parameter (*β_m_*) was assumed to vary according to the month. The transition from the latent (*E*) to the infectious (*I*) state was based on the transition rate α, where 1/α is the duration of the latency period in months.

A step of the control program represented the implementation of one or more biological tests, some of which potentially requiring the slaughter of the animal (for detecting lesions). Tests implemented in the context of a control program could be performed on the entire or part of the herd; the sample of tested animals depending on the results of previous steps of the control program. A step *k* of the control program was then represented by the quadruplet〈*Se_k_,Sp_k_,M_k_,Q_k_*〉, where:

*Se_k_* and *Sp_k_* are the joint sensitivity and specificity of tests (assumed independent of age and identical for animals in health states *E* and *I*)
*m_k_* equals 1 if the tests require the slaughter of the animal and 0 otherwise
*Q_k_* is a parameter structured according to age class and to the status of animals with respect to the step of the control program, which defines the sampling plan for testing animals: *Q_k_ (i,k)* is the probability that an animal is tested if it has age *i* and status *k* for the control program. This parameter verifies: ∑_((i,k)∈A×D)_Q*_k_* (i,*k*) = 1.

The step *k* = 1 of the control program corresponded to a screening in a bTB-free herd. The steps *k*>1 corresponded to detection and control measures conducted in a herd known or suspected to be infected.

**Figure 3:**
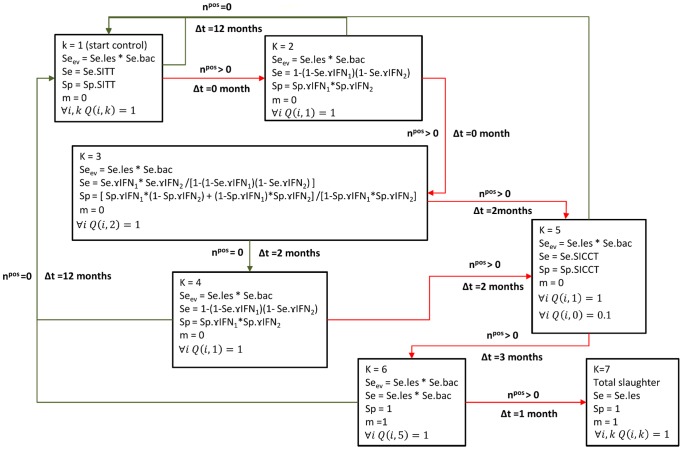
Control program C applied in the Dordogne department between 2007 and 2010. Step 1: yearly bTB screening using SITT (the herd being considered disease-free). All animals are tested (µ i,k Q(i,k) = 1). Sensitivity (Se) and specificity (Sp): those of SITT. Immediate transition to step 2 if an animal is positive (n^pos^>0). Step 2: confirmation of SITT positive results using PDD γIFN (γIFN1) and ESAT-6 γIFN (γIFN2). All the positive animals of step 1 are tested (µ i Q(i,1) = 1. Sensitivity (Se) and specificity (Sp): parallel combination of both tests (positivity to any of both tests). Immediate transition to step 3 if an animal is positive; otherwise: transition to step 1. Step 3: interpretation of the positive results of step 2 as convergent (both γIFN tests are positive) or not (only one of both tests is positive). All the positive animals of step 2 (µ i Q(i,2) = 1) are concerned. Sensitivity (Se) and Specificity (Sp): sequential combination of both tests in positive animals of step 2. Transition to step 5 if an animal is positive, and to step 4 otherwise, two months later. Step 4: second confirmation of SITT positive results using PDD γIFN (γIFN1) and ESAT-6 γIFN (γIFN2). All the positive animals of step 1 are tested (µ i Q(i,1) = 1). Sensitivity (Se) and specificity (Sp): same as for step 2. Transition to step 5 if an animal is positive, two months later; otherwise: transition to step 1, 12 months later. Step 5: confirmation of SITT positive results using SICCT. All the positive animals of step 1 are tested (µ i Q(i,1) = 1), as well as 10% of the negative animals (µ i Q(i,0) = 0.1). Sensitivity (Se) and specificity (Sp): those of SICCT. Transition to step 6 if an animal is positive, three months later. Step 6: slaughter of SICCT-positive animals and isolation of *M. bovis* from lesions. All the positive animals of step 5 (µ i Q(i,5) = 1) are slaughtered (m = 1) and bacterial culture is performed from observed lesions. Sensitivity (Se): sequential combination of a visual inspection at the slaughterhouse and of a bacterial culture. Transition to step 7 if an animal is positive, 1 month later (Δt = 1 month). Step 7: total slaughter. All the animals (µ i,k Q(i,k) = 1) are slaughtered (m = 1). Routine detection of lesions at slaughterhouse. Sensitivity (Se_ev_): sequential combination of a visual inspection at the slaughterhouse and of a bacterial culture. If a positive animal is thus detected, regardless of the current stage of control program, transition to step 7. See table 2 for the definition of the other parameters.

At each time step, it was further assumed that slaughterhouse surveillance allowed the detection of lesions in culled animals. This slaughterhouse surveillance combined the visual inspection of carcasses with the isolation of *M. bovis* from lesions (Figures 2 and 3).

### Parameterisation

#### Fixed parameters

Consistent estimates of the sensitivity and specificity of diagnostic tests are found in the literature, and herd management practices were assumed homogeneous in a given region. The values of the corresponding parameters were thus fixed according to literature or based on expert opinion.

Three batches were distinguished in beef herds: young heifers (1–2 years), heifers of reproductive age (2–3 years), and cows (after the first calving at 4 years) with calves and heifers fed by their mothers (<1year). In dairy herds, animals were also separated into three batches: young heifers (<1 year), heifers of reproductive age (1–2 years), and cows (after the first calving, at 3 years).

For both types of herds, the stabling period was between November and March. Renewal and routine cull of animals was assumed to occur between January and March, animals being culled from the age of 3 years in dairy herds and from the age of 4 years in beef herds. The maximum age of the cattle (A_max_) was set to 15 years. Other fixed parameters (culling rate, sensitivity and specificity of screening and diagnostic tests) are given in Table 2.

**Table 2:** Definitions and values of fixed parameters in the model.

Parameters	Description	Value	Range^1^	Reference
*µ_m_* (*i*)	Culling rate			(Prof. Coureau, Alfort National Veterinary School, pers. Comm.)
	Dairy herd, 2000’s	0.35	0.25; 0.45	
	Beef herd, 2000’s	0.25	0.15; 0.35	
	All herds, 1980’s	0.25		
Se.SITT	Sensitivity of single intradermal tuberculin test		0.635; 1	[8]
	1980’s	0.72		
	2000’s	0.839		
Sp.SITT	Specificity of single intradermal tuberculin test		0.755; 0.99	[8]
	1980’s	0.988		
	2000’s	0.968		
Se.SICCT	Sensitivity of single intra dermal comparativecervical tuberculin test	0.80	0.52; 1	[8]
Sp.SICCT	Specificity of single intra dermal comparativecervical tuberculin test	0.995	0.788; 1	[8]
Se.PPD γIFN	Sensitivity of gamma interferon test (PPD)	0.876	0.73; 1	[8]
Sp.PPD γIFN	Specificity of gamma interferon test (PPD)	0.966	0.850; 0.996	[8]
Se.ESAT-6 γIFN	Sensitivity of gamma interferon test (ESAT-6)	0.763	0.690; 0.836	[51]
Sp.ESAT-6 γIFN	Specificity of gamma interferon test (ESAT-6)	0.992	0.976; 1	[51]
qSICCT	Proportion of animals non-reactors to SITT testedsix weeks later by SICCT	0.10	0.05; 0.15	(French Ministry of agriculture, Food and Forestry)
Se.bac	Sensitivity of bacterial culture	0.78	0.729; 0.828	[52]
Se.les	Sensitivity of visual inspection of carcass	0.50	0.255; 0.755	[6]

1 Values used in the sensitivity analysis.

Month-specific transmission parameters allowed representing the difference between the stabling and grazing period, with *β_m_* = *β_inside_* during the stabling period (between November and March) and *β_m_* = *β_outside_* for the remainder of the year. Because of the close contact between dairy cows that occurs twice per day in the milking parlour, *β_inside_* was used throughout the year in dairy herds for the cows’ batch.

Initial conditions assumed the presence of a single infected animal in the state *E*. To represent the introduction of infection through contact during grazing, this animal was randomly selected among animals that had access to pasture (all batches for beef herds and the first two batches in dairy herds). Simulations were stopped when there were no more infected animals (thanks to routine cull and/or to the control program), the current step of the control program being 1 (i.e. screening in a bTB-free herd).

#### Parameter estimation

As estimates available in the literature are divergent or uncertain, and because many aspects of bTB pathogenesis are yet to be elucidated, three parameters were estimated from field data: *α* (transition rate between the health states *E* and *I*), *β_inside_* (transmission parameter when animals are inside the stable) and *β_outside_* (transmission parameter when animals are grazing). Individual-level data from datasets B and C were used, corresponding to 27 herds and 1423 animals (Table 1). Control programs B (Figure 2) and C (Figure 3) were modelled according to memos of the French Ministry of Agriculture, Food and Forestry.

The ABC (Approximate Bayesian Computation) method was used to estimate the parameters (*α*, *β_inside_* and *β_outside_*). This method aims at estimating the posterior distributions of the parameters where the calculation of the likelihood is difficult or burdensome [18].

The generic form of the ABC method’s “rejection algorithm” is [18]:

- 1- Sample parameter values θ* from the prior distribution π(θ).
- 2- Simulate a dataset χ* using parameter values θ*.
- 3- Calculate the summary statistics for the observed data S(χo) and simulated data S(χ*).
- 4- Compare the simulated data, S(χ*), and the observed data, S(χo), using the distance function, *d*, and the tolerance *ε*; if *d* (S(χ_o_), S(χ*)) ≤ *ε*, the value of θ* is accepted. When *ε* = 0, the posterior distribution is exactly π(θ/S (χo)), whereas when ε→+∞, the posterior distribution is equal to the prior distribution [19].

Other algorithms have been proposed for the ABC method (e.g. Markov Chain Monte Carlo or Sequential Monte Carlo). We used the rejection algorithm followed by a step of local linear regression as proposed by Beaumont et *al.* [19]. This step aims at reducing the variance of the posterior estimation in order to try to correct errors that are due to a non-zero value of the tolerance *ε*. A total of 100,000 simulations were performed for parameter estimation.

Prior distributions were uniform distributions U[0.027–0.50]month^−1^ for *α* and U [0–2]month^−1^ for *β_inside_* and *β_outside_*. For *α*, these values correspond to a period of latency between 2 and 36 months. The lower bound (2 months) is the rounded value of the lower bound (87 days) of the estimation obtained by Neill et *al*. in an experimental study [20], while the upper bound (36 months) is a rounded value of the upper bound (34 months) of the estimated duration of latency in Perez et *al*. [2]. For *β_inside_* and *β_outside_*, the upper bound of the prior distribution was chosen arbitrarily after having verified by simulation that, using this value, the median simulated percentage of animals with lesions at total slaughter (22% and 17% for dairy and beef farms, respectively) was significantly higher than the observed value in dataset B (6.4%).

The choice of summary statistics is a crucial step in the ABC method. This choice involves a compromise between information loss and size of the statistic [21]. Using a poorly chosen set of summary statistics can often lead to an overestimation of credible intervals due to the loss of information [22]. One way to capture most of the information present in the data would be to use many statistics, but the accuracy and stability of the ABC method decrease rapidly with an increasing number of summary statistics [22], [23]. In order to choose the best combination of summary statistics, eight combinations (Table 3) were tested on simulated data obtained using arbitrarily fixed parameter values. The tested summary statistics combined two types of variables, the time between the last negative control and the detection of infection and the percentage of animals with detected lesions, taking into account or not the batch, the type of herd and the year. The procedure used to select the best summary statistics among the eight tested combinations is described in Appendix S2. Briefly, parameter values were set to arbitrarily chosen values and used to generate 100 simulated datasets. In a first step, one of these datasets was chosen and considered a pseudo-observation. Each set of summary statistics was then successively used to estimate the posterior distribution of the parameters, and we kept for further analysis the set of summary statistics for which the arbitrarily chosen parameter values were inside the corresponding 95% credibility interval. In a second step, the preceding procedure was successively applied to the 100 simulated datasets and we selected the set of summary statistics that maximized the proportion of cases for which the arbitrarily chosen parameter values were inside the 95% credibility interval of the posterior distribution of parameters.

**Table 3:** Selection of the set of summary statistics based on simulated datasets generated using arbitrarily fixed parameter values: α = 0.083 months, β_inside_ = 0.5, and β_inside_ = 0.1.

Id	Summary statistics	Dimension	Median [95% credible interval]			p^1^
			α	β_inside_	β_outside_	
A	Average percentage of animals with detected lesions^2^	1	0.72 [0.60–0.78]	0.79 [0.44–1.27]	0.61 [0,03–1,92]	–
B	Average percentage of animals with detected lesions in each batch	3	0.42 [0.00–2.43]	0.33 [0.28–0.70]	0.29 [0.00–3.9]	73%
C	Percentage of animals with detected lesions in each batch (n = 3)of each herd (n = 27)	81	0.13 [0.06–0.67]	0.35 [0.02–1.97]	0.44 [0.01–2.21]	49%
D	Same as C+percentage of herds submitted to total slaughter	82	0.97 [0.48–2.04]	0.05 [0.01–0.15]	0.03 [0.00–0.12]	–
E	Same as C+time between the last negative test and the detectionof infection for each herd	108	0.29 [0.07–0.69]	0.35 [0.00–0.77]	0.07 [0.00–0.84]	32%
F	Same as E+percentage of herds submitted to total slaughter	109	0.33 [0.12–0.65]	0.29 [0.08–0.57]	0.08 [0.00–0.79]	–
G	Average percentage of animals with detected lesion for each batch,period^3^ and herd type^4^; average time between the last negativetest and the detection of infection for each period and herd type	16	0.26 [0.07–0.74]	0.27 [0.04–0.75]	0.07 [0.00–0.52]	71%
H	Same as G+percentage of herds submitted to total slaughterfor each period and herd type	20	0.073 [0.05–0.54]	0.48 [0.03–0.76]	0.12 [0.00–0.59]	88%

1 Proportion of cases for which each of the three arbitrarily fixed parameter values were inside the 95% credible interval of the posterior distributions (100 repetitions).

2 For herds submitted to a total slaughter.

3 Before 2007 (control program B) and after 2007 (control program C).

4 Dairy of beef.

The tolerance threshold *ε* was determined by choosing the proportion of retained simulations p_ε_. A leave-one-out procedure was applied to a set of 100 datasets simulated using parameters (*α, β_inside_, β_outside_*) sampled from the prior distributions, to evaluate the robustness of the estimates to the tolerance rate. Each of the 100 simulated datasets was successively considered a pseudo-observation and parameter values were estimated using the above procedure. A prediction error coefficient was calculated for each estimated parameter, for values of p_ε_ ranging from 0.1% to 5% [24]. The smallest value of p_ε_ for which the prediction error coefficient was stable for the three parameters (*α, β_inside_, β_outside_*) was thus selected.

We used the “abc” package of software R [25]. The function cv4abc was used to select the value for the proportion of retained simulations p_ε_.

A sensitivity analysis was performed to evaluate the effect of changing the fixed parameter values (Table 2) on the estimated values of the transition rate (*α*) and of the transmission parameters (*β_inside_* and *β_outside_*). Two different values were selected for each fixed parameter (Table 2) according to literature (SITT, SICCT, γIFN and bacterial culture) or using fixed deviations (±10% or ±5%). Only first-order effects were analyzed and, for each scenario, *α*, *β_inside_* and *β_outside_* were estimated by the ABC method, as described above. This operation was repeated 24 times (12 fixed parameters and 2 points per parameter) resulting in 24 posterior distributions for *α*, *β_inside_* and *β_outside_*. These were analysed using three generalised linear model (GLMs) (one per estimated parameter). The explanatory variables were the fixed parameters values (their deviation from their default values).

The GLMs were used to predict the effect of an increase of 5% of each fixed parameter on the average values of *α*, *β_inside_* and *β_outside_*. To identify the most influential fixed parameters, we compared the coefficient of variation of the posterior distributions (obtained using the default values of fixed parameters) with the relative error induced by a 5% change of fixed parameters, as predicted by the GLMs.

### Validation

An internal validation was first performed using a leave-one-out cross-validation procedure. One hundred triples of parameter values (*α*, *β_inside_* and *β_outside_*) were randomly chosen among the 100,000 used for parameter estimation. Each was successively used to generate a dataset considered a pseudo-observation, and posterior distributions were estimated using the above procedure (without using the pseudo-observation). The reliability of the estimation procedure was quantified by the proportion of cases for which the triple (*α*, *β_inside_* and *β_outside_*) used to generate the dataset was within the 95% credible interval.

An external validation was performed to test the ability of the model to reproduce the observational data of bTB in France between 1980 and 2010, using the data that were not used for parameter estimation: dataset A, aggregated data from datasets B and C, and dataset D (Table 1). Each herd of these datasets was successively used to parameterize the model (herd size and type, control program - Figures 2, 3, S1 and S2), and the posterior distributions of parameters (*α*, *β_inside_* and *β_outside_*) were sampled to perform 1000 simulations. Those ending by a total slaughter were kept to compute the distribution of the proportion of animals with detected lesions. For each herd of the above datasets, the observed proportion was compared with the simulated distribution thus obtained. Furthermore, for dataset A, the predicted proportion of simulations ending by a total slaughter was compared with the percentage observed in the corresponding department in the 1980s: 5%.

### Model exploitation

The basic reproduction ratio (R0) is used to measure the transmission potential of a disease. It is the number of secondary infections produced by an infectious animal in a fully susceptible population that is totally susceptible. This indictor is of great importance in infectious disease modelling as, when R0<1, the disease tends to vanish, whereas when R0>1, the disease tends to spread and a large epidemic may occur [26]. However, in real situations, the population is rarely entirely susceptible, mainly because of the spread of infection (and the corresponding decrease in the number of susceptible individuals). We computed the effective reproductive ratio R(t): the average number of cases secondary to an infectious case in a population consisting of susceptible and infected individuals. Two average herds, respectively representative of a French dairy herd (81 cattle) and a French beef herd (70 cattle) in the 2000s, were considered. Disease dynamics was simulated in these herds without a control program, and the distribution of the effective reproductive ratio R(t) was calculated each month after the introduction of *M. bovis*, during 360 months (twice the maximal age of cows in the model: 15 years). Details of R(t) calculation are given in Appendix S3.

## Results

### Parameter estimation

The procedure used to choose the set of summary statistics first led to select B, C, E, G and H, for which, when using one simulated dataset as a pseudo-observation, the arbitrarily chosen parameter values were all inside the 95% credible interval of posterior distributions of parameters (Table 3). Their dimensions varied between 3 for B and 109 for F (Table 3). Among these five sets of summary statistics, H maximized the proportion of cases (88%) where the arbitrarily fixed parameters (*α*, *β_inside_* and *β_outside_*) were all within the credible interval of 95% of posterior distributions when using the 100 simulated datasets as pseudo-observations (Table 3). This set of summary statistics was thus chosen: a combination of statistics describing the percentage of animals with detected lesions per batch and the percentage of herds with total slaughter per herd type (dairy/beef) and per period (periods where control program B and C were applied). The evolution of the values of the prediction error coefficient according to the proportion of retained simulations led to select p_ε_ = 2%.

**Figure 4:**
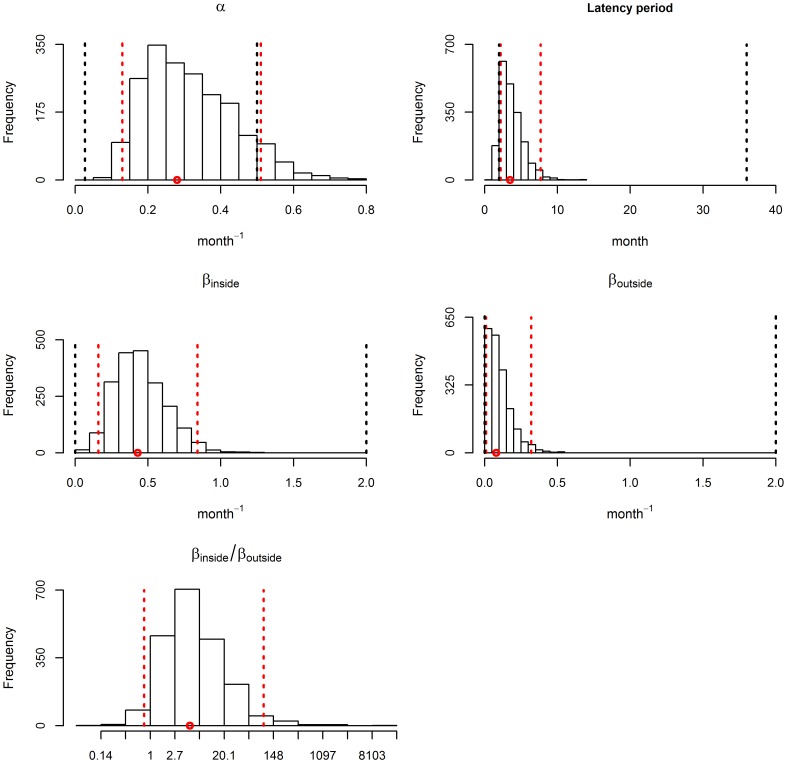
Posterior distribution of parameters *α*, *β_inside_* and *β_outside_*. Red dots: median value of parameter estimates; black dotted lines: lower and higher bounds of parameter prior distributions; red dotted lines: 95% credible intervals of the parameter posterior distributions. Histogram β_inside_/β_outside_ represents the value of log ratio β_inside_/β_outside_ but the x-axis values are not expressed in log.

**Figure 5:**
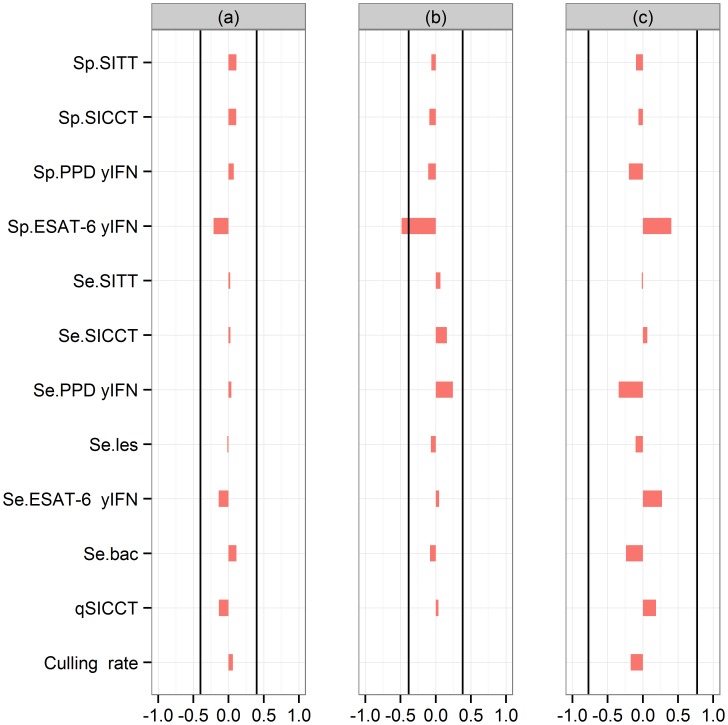
Impact of changes of fixed parameter values on the estimates of *α*, *β_inside_* and *β_outside_*. (a): relative error for α; (b): relative error for β_inside_; (c): relative error for β_outside_; black line: value of the coefficient of variation of the posterior distributions obtained when we using the default values of fixed parameters; if the relative error value is within the two black lines, the effect of fixed parameters values on parameter estimates in the model is considered limited; qSICCT: percentage of SITT-negative animals tested 6 weeks after by SICCT; see table 2 for the definition of the other parameters.

The median of the posterior distribution of *α* was 0.28 (95% credible interval [CI]: [0.13–0.56]), which corresponds to a median latency period for bTB of 3.5 months (95% CI: [2]–[8] months) (Figure 4). The median value of bTB transmission coefficients were 0.43 month^−1^ (95% CI [0.16–0.84] month^−1^) inside the stable (*β_inside_*) and 0.08 month^−1^ (95% CI [0.01–0.32] month^−1^) outside the stable (*β_outside_*) (Figure 4). The median of the ratio *β_inside_*/*β_outside_* was 5 (95% CI [0.8–100]). It was>1 in 95.6% of cases (Figure 4). Negative values of Spearman’s rank correlation coefficient were observed between *α* and *β_inside_* (−0.47, p<0.0001), between *α* and *β_outside_* (−0.31, p<0.0001), and between *β_inside_* and *β_outside_* (−0.35, p<0.0001). These negative correlations were expected, as the summary statistics (such as the proportion of animals showing lesions) may remain approximately the same if a higher value of α (i.e. a shorter latency period) is compensated by smaller values of *β_inside_* and *β_outside_*, or if a higher value of *β_inside_* is compensated by a lower value of *β_outside_*. The results of the sensitivity analysis of parameter estimates to variations of fixed parameter values are summarised in a tornado chart (Figure 5). A 5% increase in the values of the fixed parameters had limited effects on the estimation of *α* and *β_outside_* (Figure 5). For *β_inside_*, the relative difference of the average posterior estimate greater than the coefficient of variation computed from the posterior distribution of for a single parameter: the specificity of the ESAT-6 γIFN test. A 5% increase of the specificity of the ESAT-6 γIFN test led to a 48.5% decrease of the average posterior estimate of *β_inside_*.

### Predicted bTB dynamics

**Figure 6:**
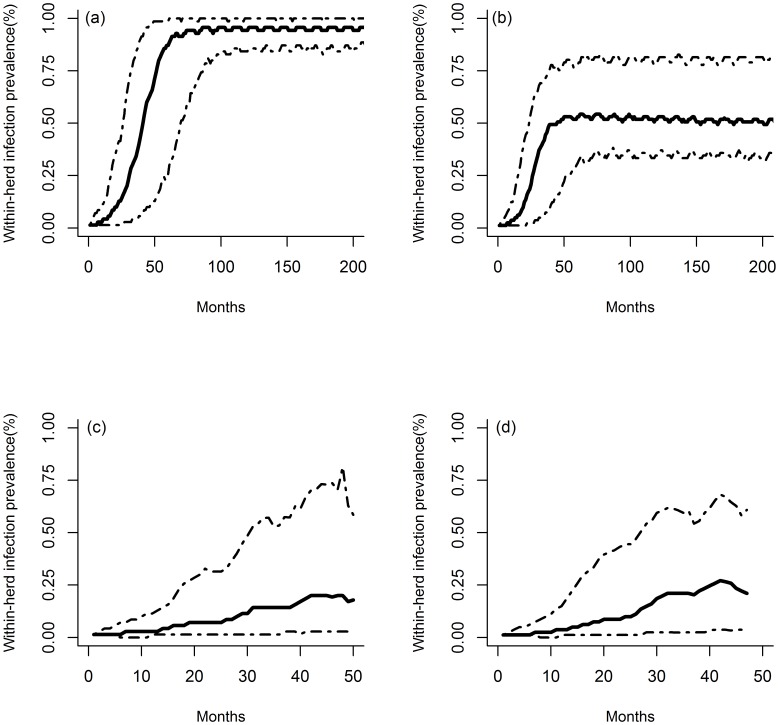
Monthly evolution of the simulated proportion of infected animals (animals in E or I health state; solid line: median, dashed: 2.5% and 97.5% percentiles) in standard French beef (a and c, 70 animals) and dairy (b and d, 81 animals) herds without (a and b) or with (c and d) implementation of control program B (1,000 simulations per scenario).

**Figure 7:**
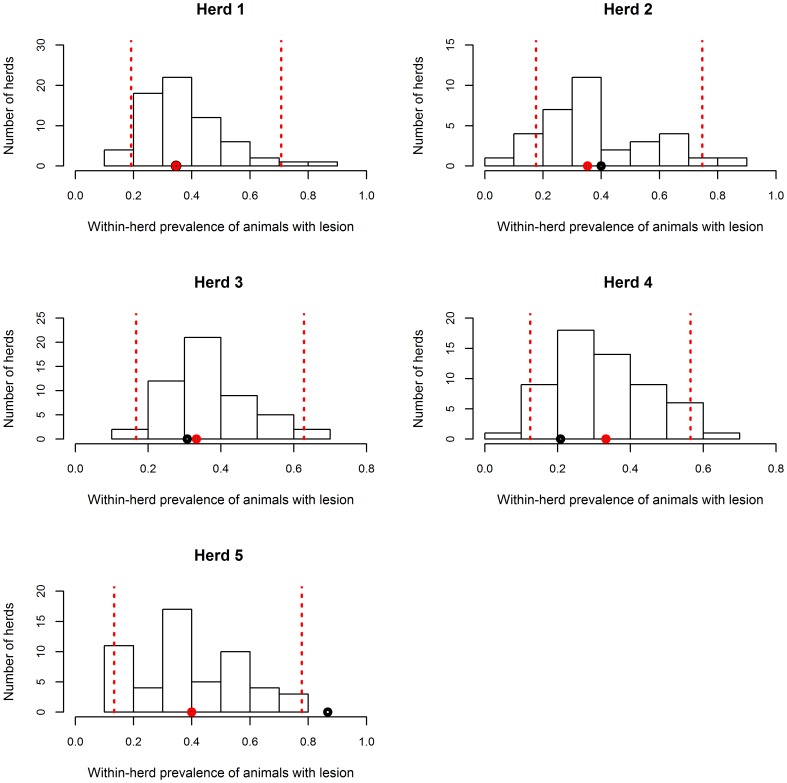
Observed and simulated proportions of animals with detected lesions when total slaughter is performed, for the 5 herds of dataset A. Black point: observed proportion of animals with bTB-like lesions in each herd; red point: simulated median proportion of animals with bTB-like lesions in each herd; dotted lines: 2.5% and 97.5% percentiles of the simulated distributions.

Figure 6 and Table 4 represents the predicted bTB dynamics in two herds representing dairy farms (average size in 2000∶81 animals) and beef herds (average size in 2000∶70 animals) in France, with and without a control program (control program B, Table 1 and Figure 2). When the control program was simulated, after its introduction in a susceptible herd, bTB was predicted to disappear in 22% of cases in beef herds and in 17% of cases in dairy farms, thanks to routine cull: the median time from disease introduction and disease extinction was less than 10 months, in both types of herds, with or without a control program (Table 4). In most cases, *M. bovis* did not disappear from the simulated herds and the disease was predicted to be detected through passive surveillance (at slaughterhouse) (13% for beef herds and 21% for dairy farms) or through active surveillance (screening using skin tests) (65% for beef herds and 62% for dairy farms). In both cases, the median time period between the introduction of infection and total slaughter was less than 3 years in the two types of herds (Table 4). In the absence of a control program (Figure 6a and 6b), the median proportion of infected animals was predicted to increase until reaching a plateau at 61 months in beef herds and 40 months in dairy herds (Figure 6a and 6b). The corresponding level of infection prevalence was predicted to be higher in beef herds (Figure 6a) than in dairy herds (Figure 6b): once reached the enzootic infection level the median prevalence of infected animals was 93% in beef farms and 53% in dairy herds. As expected, when a control program was used, the maximum percentage of infected animals was predicted to be lower in both types of herds (Figure 6c and 7d).

**Table 4:** Predicted median and percentiles [2.5%–97.5%] of the distribution of outbreak duration (in months) in standard French beef herds (70 animals) and dairy herds (81 animals) without or with control programs (1,000 simulations per scenario).

		Beef herds	Dairy herds
Time to natural extinction of infection	Without a simulated control program	7 [3]–[30]	8 [4]–[32]
	With a simulated control program		
	No detection of infection	6 [3]–[11]	7 [4]–[16]
	Infection suspected but not confirmed^1^	8 [3]–[25]	8 [3]–[23]
Time to total slaughter of the herd after infection detectionby routine skin testing		24 [9]–[50]	23 [9]–[48]
Time to total slaughter of the herd after infection detectionby slaughterhouse surveillance		24 [8]–[50]	30 [9]–[47]

1 No isolation of *M. bovis*, total slaughter is thus not applied.

### Validation

Internal validation showed that, for 83 of the 100 randomly selected simulations, the fixed values of the three parameters *α*, *β_inside_* and *β_outside_*, were inside the 95% credible interval of the posterior distributions.

Using dataset A, external validation showed that, in four of five herds (farms 1, 2, 3 and 4), the observed percentage of cattle with lesions was between the 2.5% and 97.5% percentiles of the simulated proportion of animals with lesions at total slaughter (Figure 7). In the fifth herd, the observed percentage of animals with lesions was very high (86.6%) and was above the 97.5% percentile of the simulated distribution (Figure 7). The proportion of simulations ending with a total slaughter varied between 3% and 7%, depending on the herd. These values are close to the proportion of 5% reported by the French Ministry of Agriculture, Food and Forestry for that department at that time.

The observed percentage of animals with lesions was located between the 2.5% and 97.5% percentiles of the simulated proportion of animals with lesions in 92% (23 herds out of 25) and 83% (24 herds out of 29) of herds of datasets B and C, respectively. This percentage was 100% (29 herds out of 29) for dataset D.

### Effective reproductive ratio

**Figure 8:**
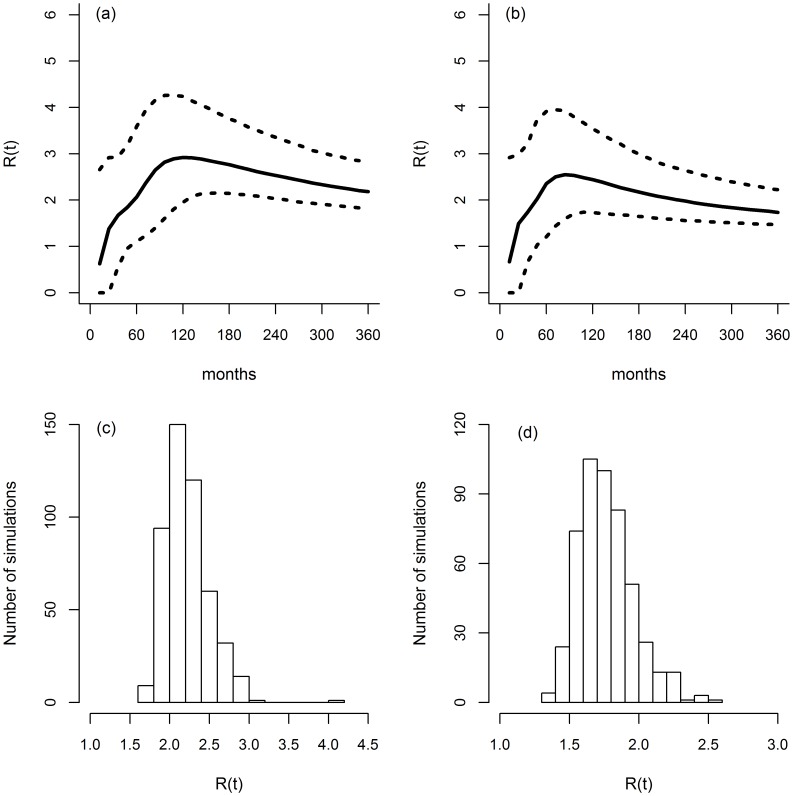
Monthly evolution of the simulated effective reproductive ratio R(t) (a and b), and distribution of this R(t) (c and d) when the disease has become enzootic (after 30 years) in standard French beef (a and c, 70 animals) and dairy (b and d, 81 animals) herds. Solid line: median; dashed: 2.5% and 97.5% percentiles.

Figures 8a and 8b show the evolution of R(t) values over time. In both types of herds, the shape of the curve was similar, with an increase in the values of R(t) that reached a plateau 72 months after disease introduction in dairy herds and at 96 months after disease introduction in beef herds. After 30 simulated years, which corresponds to a situation of enzootic circulation of *M. bovis* within the herd, the value of R(t) was slightly higher for beef herds than for dairy herds. The median value and the percentiles [2.5%–97.5%] of the predicted distribution of R(t) were 2.2 [1.8–2.8] for beef herds and 1.7 [1.5–2.2] for dairy herds (Figure 8a and Figure 8b). It was higher than 1 for each of the 1,000 simulations and higher than 2 for 11% of simulations in dairy herds and 79% in beef herds (Figure 8c and Figure 8d).

## Discussion

In this work, we have built a stochastic discrete-time model to simulate bTB dynamics and control in cattle herds. The model accounts for interactions among the three processes that influence the within-herd evolution of bTB: (i) the natural history of the disease (infectious process), (ii) husbandry practices (demographic process) and (iii) control programs (detection and control process). The model has been designed to allow simulating control programs of arbitrary degree of complexity. Parameterization allows representing various herd types and farming practices (in particular the differences between dairy herd and beef herd management). Epidemiological parameters of the within-herd transmission of bTB (duration of the latent state, transmission parameters) were estimated using a first set and the model was validated using a second set of field data collected between 1980 and 2010. Herd management practices and bTB control programs have evolved during this period and the model could be successfully adapted by changing parameter values, suggesting a good level of genericity and an ability to simulate various herd management systems and bTB detection and control programs of arbitrary complexity. The simulated effective reproductive ratio R(t) may allow assessing the effectiveness (*i.e.* whether R(t)<1) of different bTB control strategies or the effect of changes in herd management practices on the bTB within-herd dynamic.

Internal validation showed a good reliability of the estimation procedure of model parameters by the ABC method. External validation showed that the model was able to reproduce field data regarding bTB collected between 1980 and 2010 in several French departments. This suggests that the transmission coefficients (*β_inside_* and *β_outside_*) and the duration of the latent period (1/*α*) have remained roughly constant between the 1980s and today, despite changes in parameters related to herd management practices such as the culling rate, the age at culling and the herd size.

Health states used in the proposed model (susceptible, latent and infectious) are consistent with the pathology of infection. However, published models often divide the latent state into two sub-states (latent, non-skin test-responsive and latent, skin test-responsive [13]–[15]) and use shorter time steps (day or week). Latent non-skin test-responsive animals spend an average of four weeks in this state before developing a positive skin test reaction [13], [14], [27]. Conlan et *al*. [28] estimated the median period of the latent non-skin test-responsive state at 28 days. As we used a monthly time step, it was thus not necessary to represent this health state. Infectious state *I* was defined by the presence of detectable lesions at slaughter. The type (open or stabilised) and the location of the lesions were not taken into account. This may induce an underestimation of within-herd transmission: an animal with so-called “open tuberculosis lesions” may infect the rest of the herd in a short period of time. However, much is unclear about open tuberculosis lesions, including the probability of developing this type of lesion, its duration and its infectivity [15]. Therefore we did not incorporate the type of lesion in our model. This choice is in line with other epidemiological models of bTB [13]–[15].

Most of the models previously developed in the literature that simulated the within-herd spread of bTB used a single transmission parameter. In our model, we used two transmission parameters to distinguish transmission inside buildings and in pastures. Both transmission coefficients are related to herd practices, allowing to use the model for representing various farming systems by changing the grazing periods and the composition of batches. The within-herd transmission of bTB is considered higher in dairy herds than in beef herds [12]. This is attributed to a combination of several factors that contribute to the spread of infections in dairy herds such as the high contact rate, especially in the milking parlour [13], the high animal density and stress factors related to intense animal management [12], [29]–[32]. The estimated value of *β_inside_* was greater than that of *β_outside_* in 96% of cases, suggesting that the definition of two transmission coefficients allowed capturing the difference between dairy herds and beef herds in terms of within-herd transmission of bTB. Indeed, although the 95% credible intervals of the two within-herd transmission coefficients of bTB (*β_inside_* and *β_outside_*) overlapped, the median within-herd transmission coefficient inside the stable was 5 time as high as in the pasture: 0.43 month^−1^ [0,16–0.84] and 0.08 month^−1^ [0.01–0.32] for *β_inside_* and *β_outside_,* respectively. These estimated medians were consistent with the estimates of within-herd transmission of bTB in Spain proposed by Alvarez et* al*. [12], with higher transmission coefficients in dairy herds (median 0.39 month^−1^) than in beef herds (0.19 month^−1^). The estimated values of the transmission coefficients are also in the same range as values obtained by other studies: 0.22 month^−1^ in the study of Barlow et *al*. [13] and 0.18 months^−1^ in the study of Perez et *al.* in Argentina [2].

The only transmission route taken into account in our work is the direct transmission between animals of the same batch. Indirect transmission, the potential role of wildlife, contacts with cattle from other herds at pastures and contacts between cattle of different batches are not taken into account. In particular, between-batch disease transmission was not taken into account because we assumed that the probability of contact between animals of different batches was low: different batches of the same herd are often placed by the breeder on different and distant pastures and even sometimes in separate buildings. It should be noted that the transmission between batches does nevertheless occur in the model: as bTB is a chronic, slowly evolving disease, between-batch transmission occurs when animals are moved from one batch to another. However, the closed herd assumption would likely underestimate the persistence of *M. bovis* infection in herds, if wildlife or introduced animals can reintroduce infection [33].

The median duration of the estimated latency period (state *E*) was 3.5 months [2]–[8] months. This range of values is consistent with the chronic nature of bTB. However, our estimate of the latency duration was lower than the mean estimated duration of 24 months (95% confidence interval: 15–34 months) that was obtained by a simulation model of the within-herd transmission of bTB in Argentina [2]. This difference may be explained by differences among animals in the two countries, as the incubation period depends on the susceptibility of the host [34].

Model predictions for 2000 showed that the infected herds (dairy or beef) were in 15 to 20% of cases detected by slaughterhouse surveillance. This result was expected because the frequency of intradermal skin test applied in control program B was biennial. The percentage of spontaneous extinction of the disease (thanks to routine cull) was almost identical between dairy and beef herds.

When no control program was applied, simulated within-herd prevalence reached>50% in both types of farms. This simulated prevalence is high compared to the values observed in some parts of Africa and Asia (between 6% and 15%), where no control program is applied [35], [36]. In Africa and in Asia, most cattle are zebu (*B. indicus*) [37]. Ameni *et* *al*. [38] report that, under identical field husbandry conditions, a higher susceptibility to bTB was observed in Holstein cows than in zebus: (odds ratio: 2.32). In France dairy cattle are kept most of the time indoors, while beef cattle have stabling periods of approximately five months each year. Oppositely, in Africa and in Asia, cattle are most of the time kept outdoors, where contacts between animals are less intense than indoors, hence the lower observed within-herd prevalence in these regions.

The low predicted values of the effective reproductive ratio R(t) over time are consistent with field observations of a low bTB prevalence in French infected herds in the 2000s, and support the results of transmission trials indicating that cattle do not readily infect other cattle [39], [40]. According to our estimation, when no control program is applied and once bTB has become enzootic within the herd, the median number of animals infected by an infectious animal is 2.2 in beef herds and 1.7 in dairy herds. This difference is due to a lower age at culling for dairy cows than for beef cows.

Several models have been proposed in the literature to simulate the dynamic of within-herd transmission of bTB: stochastic Reed-Frost model in discrete time [2], [12], Reed-Frost model in continuous time [14], stochastic individual based model [15], compartmental stochastic model in continuous time [28] and compartmental stochastic model in discrete time [13], [33], [41]. We used a compartmental stochastic model operating in discrete time. Bovine TB health states are best represented using discrete health states (S-E-I), we thus chose to use a compartmental model. The scarcity and the discrete nature of the main simulated event which is the infection of animals (in a national survey in France between 2005 and 2007 in infected herds, the median of animals with lesion was 2), led us to use a stochastic approach rather than a deterministic approach. ABC methods are computationally cumbersome. Discrete time simulation was chosen as it appeared less computationally demanding than continuous time simulation. The duration of the time step (one month) was chosen because in literature the estimated duration of the latent state (in models with a single latent state [2] or with two states of latency [28]) was greater than one month. In addition, for the modeled screening, control and demographic processes, a one month time step also appeared satisfactory (frequency of tuberculin test, stabling period).

While this type of model is relatively easy to simulate, it is analytically difficult to manage when the number of states is high. In this case, numerical resolution by simulation is the only way to estimate the parameters [42]. The likelihood is difficult to calculate in our model, because the infection process is only partially observed (only a fraction of infectious animals is detected at the slaughterhouse, depending on the sensitivity of the visual inspection of carcasses). We thus chose to use the ABC method for the estimation of model parameters because this method does not require the calculation of the likelihood [43]. The choice of summary statistics and of the tolerance threshold are two sources of error in the ABC method [44]. To reduce errors linked to the choice of summary statistics, a preliminary step was performed in which several summary statistics were tested and compared using simulated data. Other methods have been proposed in the literature such as the algorithm developed by Barnes et *al*. [45] to choose a set of summary statistics from all possible summary statistics. This algorithm uses the mutual information as a selection tool [45]. Choosing a non-zero value for tolerance, *ε*, can bias the posterior estimation of the parameters [44]. To reduce errors due to non-zero values of *ε*, the local linear regression algorithm of the ABC method was used in our work for estimating the model parameters [19]. ABC-SMC (Sequential Monte Carlo) algorithm allows using specific methods to select the tolerance threshold [46], [47]. But, according to Filippi et* al*. [48], more work needs to be done on the choice of tolerance threshold in the ABC-SMC algorithm. Besides, the use of the local linear regression algorithm is however not without problems, as the relationship between summary statistics and parameters is highly non-linear in most cases, which may bias the estimation of parameters [19].

The sensitivity analysis of a model is of great interest [42] because it helps to determine the most influential parameters. The sensitivity analysis performed here was used to independently assess the effect of fixed parameters values on the posterior parameter estimates. For practical reasons (of computation time required to perform this sensitivity analysis), the sensitivity analysis did not assess the effect of interactions among fixed parameters. Nevertheless, according to the literature, this method is the first step in exploring global associations (between the estimated and non-estimated parameters in the model), excluding interactions [49]. The results of the sensitivity analysis showed that fixed parameters have only limited effects on the posterior parameter estimates, except for the effect of the specificity of ESAT-6 γIFN test on *β_inside_*. The specificity of ESAT-6 γIFN test is used in control program C (Figure 3), which includes a 2-steps confirmation of positive SITT using γIFN test (steps 2 and 3 in Figure 3). When the specificity of ESAT-6 γIFN is increased to 100% (see Table 2), the overall specificity of the 3^rd^ step of control program C also reaches 100% (see Figure 3). Because the overall sensitivity of both γIFN-based confirmation steps is low, the time required to detect bTB increases, which induces a decrease of *β_inside_* estimate.

We assumed that the sensitivity and the specificity of the screening tests were independent of cattle age. Proaño-Perez et *al*. [50] reported a negative correlation between the age of cattle and the response to avian tuberculin. This result suggests that young animals are more likely to show false positive reactions to SITT than adult animals. The impact on our results is however likely to be limited, as the sensitivity analysis showed that the specificity of screening test had a little effect on the estimated values of the epidemiological model parameters (α, β_inside_ and β_outside_).

In conclusion, the model proposed here has been designed to be generic enough to allow simulating various herd management systems and bTB detection and control programs. Its parameters have been estimated using field data and it has been validated using an independent dataset. In the future, this model will be used to analyse the impacts of changes of control programs and of herd management practices on the dynamics of bTB in France, between the beginning of the control program (in 1965) and obtaining the bTB-free status (in 2000). Besides, it will be used to assess the effectiveness of bTB control programs, in order to identify alternative strategies to the total slaughter protocol currently applied in infected herds.

## Supporting Information

Figure S1 Control program A applied in the Nord department between 1981 and 1983. Step 1: yearly bTB screening using SITT (the herd being considered disease-free). All animals are tested (µ i,k Q(i,k) = 1). Sensitivity (Se) and specificity (Sp): those of SITT. Transition to step 2 if the proportion of positive animals exceeds a predefined threshold (n^pos^/n^test^>P.ab), one month later (Δt = 1 months). Transition to step 3 if positive results are observed, the proportion being below the threshold. Step 2: total slaughter. All the animals (µ i,k Q(i,k) = 1)are slaughtered (m = 1). Step 3: selective slaughter. Positive animals of step 1 (µ i Q(i,1) = 1)are slaughtered (m = 1). Transition to step 1, 6 months later. See table 2 for the definition of the other parameters.
(TIF)

Figure S2 Control program D applied in the Côte d’Or department between 2005 and 2009. Step 1: biennial bTB screening using SICCT (the herd being considered disease-free). All animals are tested (µ i,k Q(i,k) = 1). Sensitivity (Se) and specificity (Sp): those of SICCT. Immediate transition to step 2 if non-negative results are observed (n^pos^>0). Step 2: interpretation of SICCT non-negative results. All the positive animals of step 1 (µ i Q(i,1) = 1) are concerned. Sensitivity: for an infected animal, probability that a non-negative positive result is not doubtful (pi: probability of a doubtful SICCT result for animals in health states E or I); specificity: for a susceptible animal, probability that a non-negative SICCT result is doubtful (ps: probability of a doubtful SICCT result for animals in health state S). Transition to step 3 if all the non-negative SICCT animals are doubtful, 2 months later; otherwise: transition to step 4, 3 months later. Step 3: confirmation of the positive results of step 1 using SICCT. All the positive animals of step 1 are tested (µ i Q(i,1) = 1), as well as 10% of the negative animals (i Q(i,0) = 0.1). Sensitivity (Se) and specificity (Sp): those of SICCT. Transition to step 5 if an animal is positive, three months later; otherwise: transition to step 1. Step 4: slaughter of the positive animals of step 2 and isolation of *M. bovis* from lesions. All the positive animals of step 2 (µ i Q(i,2) = 1) are slaughtered (m = 1) and bacterial culture is performed from observed lesions. Sensitivity (Se): sequential combination of a visual inspection at the slaughterhouse and of a bacterial culture. Transition to step 6 if an animal is positive, 1 month later (Δt = 1 month). Step 5: slaughter of the positive animals of step 3 and isolation of *M. bovis* from lesions. All the positive animals of step 3 (µ i Q(i,3) = 1) are slaughtered (m = 1) and bacterial culture is performed from observed lesions. Sensitivity (Se): sequential combination of a visual inspection at the slaughterhouse and of a bacterial culture. Transition to step 6 if an animal is positive, 1 month later (Δt = 1 month). Step 6: total slaughter. All the animals (µ i,k Q(i,k) = 1) are slaughtered (m = 1). Routine detection of lesions at slaughterhouse. Sensitivity (Se_ev_): sequential combination of a visual inspection at the slaughterhouse and of a bacterial culture. If a positive animal is thus detected, regardless of the current stage of control program, transition to step 6. See table 2 for the definition of the other parameters.
(TIF)

Appendix S1 Model equations.
(DOCX)

Appendix S2 Choice of summary statistics.
(DOCX)

Appendix S3 Effective reproductive rate R(t).
(DOCX)
