# Supplementary figures and images for: Modeling Tuberculosis Dynamics, Detection and Control in Cattle Herds
Source: PLoS One. 2014 Sep 25;9(9):e108584. doi: 10.1371/journal.pone.0108584 (PMC4177924; doi:10.1371/journal.pone.0108584)

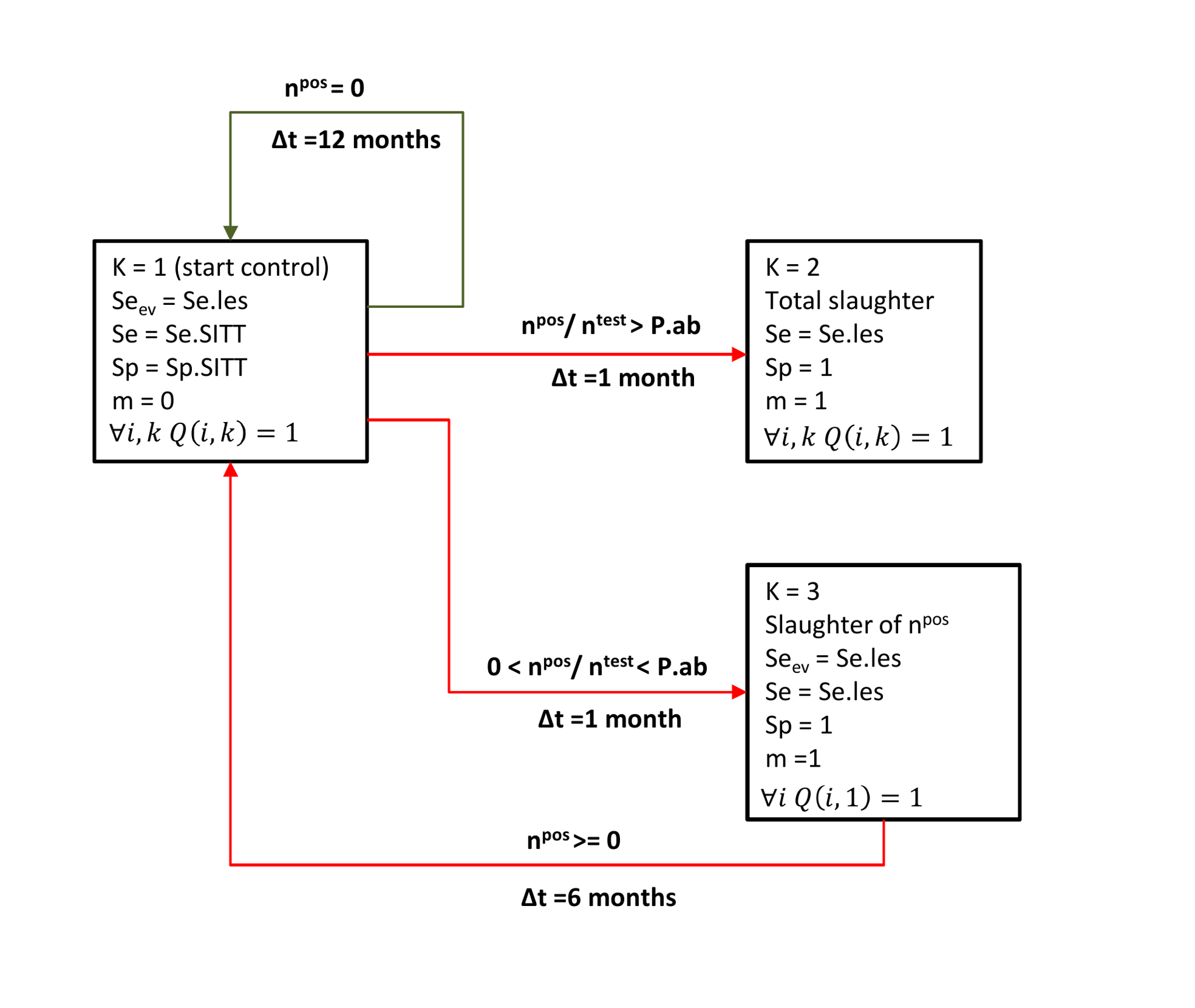

Supplement: Figure S1 — Control program A applied in the Nord department between 1981 and 1983. Step 1: yearly bTB screening using SITT (the herd being considered disease-free). All animals are tested (µ i,k Q(i,k) = 1). Sensitivity (Se) and specificity (Sp): those of SITT. Transition to step 2 if the proportion of positive animals exceeds a predefined threshold (npos/ntest>P.ab), one month later (Δt = 1 months). Transition to step 3 if positive results are observed, the proportion being below the threshold. Step 2: total slaughter. All the animals (µ i,k Q(i,k) = 1)are slaughtered (m = 1). Step 3: selective slaughter. Positive animals of step 1 (µ i Q(i,1) = 1)are slaughtered (m = 1). Transition to step 1, 6 months later. See table 2 for the definition of the other parameters. (TIF) [file pone.0108584.s001.tif]

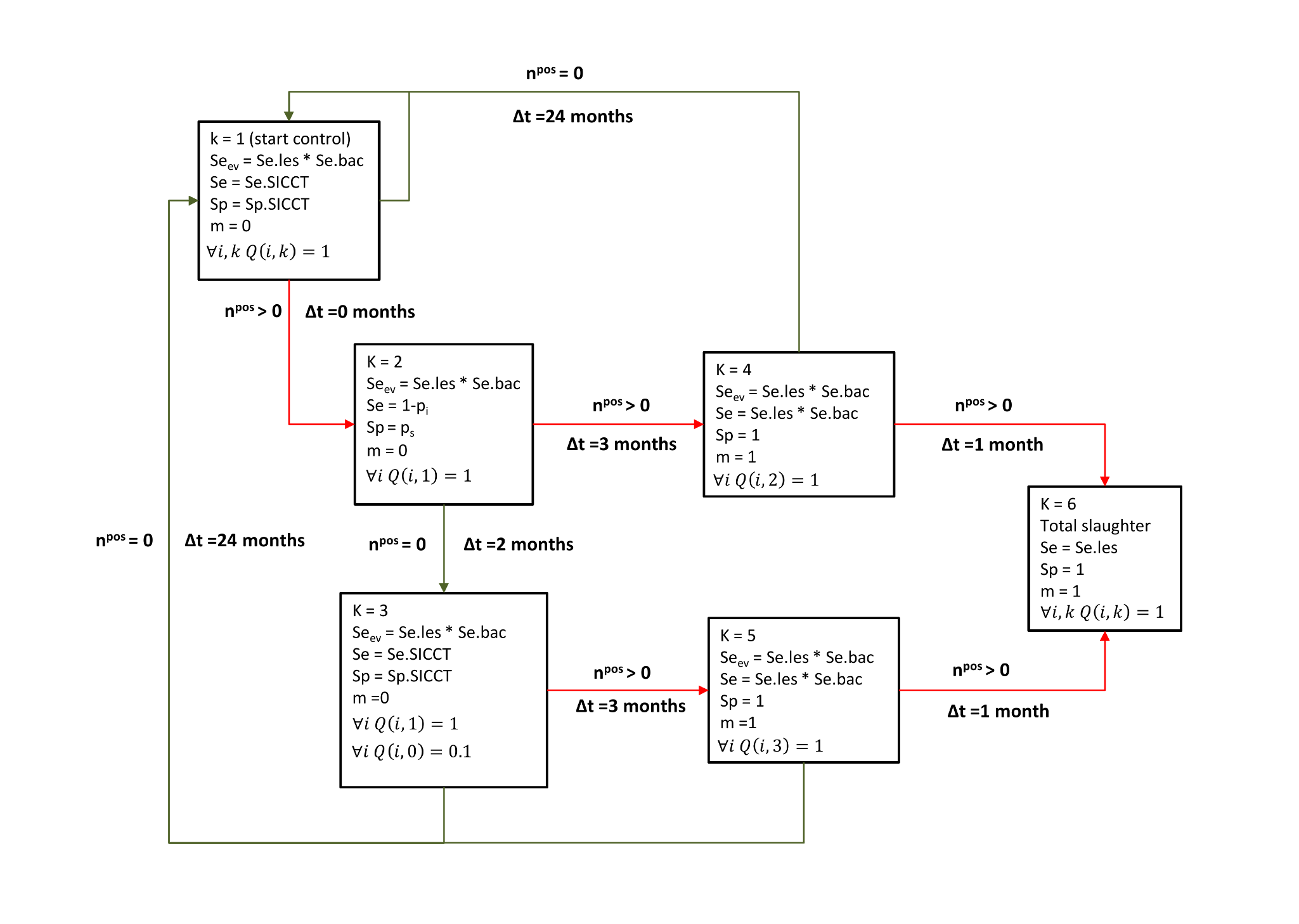

Supplement: Figure S2 — Control program D applied in the Côte d’Or department between 2005 and 2009. Step 1: biennial bTB screening using SICCT (the herd being considered disease-free). All animals are tested (µ i,k Q(i,k) = 1). Sensitivity (Se) and specificity (Sp): those of SICCT. Immediate transition to step 2 if non-negative results are observed (npos>0). Step 2: interpretation of SICCT non-negative results. All the positive animals of step 1 (µ i Q(i,1) = 1) are concerned. Sensitivity: for an infected animal, probability that a non-negative positive result is not doubtful (pi: probability of a doubtful SICCT result for animals in health states E or I); specificity: for a susceptible animal, probability that a non-negative SICCT result is doubtful (ps: probability of a doubtful SICCT result for animals in health state S). Transition to step 3 if all the non-negative SICCT animals are doubtful, 2 months later; otherwise: transition to step 4, 3 months later. Step 3: confirmation of the positive results of step 1 using SICCT. All the positive animals of step 1 are tested (µ i Q(i,1) = 1), as well as 10% of the negative animals (i Q(i,0) = 0.1). Sensitivity (Se) and specificity (Sp): those of SICCT. Transition to step 5 if an animal is positive, three months later; otherwise: transition to step 1. Step 4: slaughter of the positive animals of step 2 and isolation of M. bovis from lesions. All the positive animals of step 2 (µ i Q(i,2) = 1) are slaughtered (m = 1) and bacterial culture is performed from observed lesions. Sensitivity (Se): sequential combination of a visual inspection at the slaughterhouse and of a bacterial culture. Transition to step 6 if an animal is positive, 1 month later (Δt = 1 month). Step 5: slaughter of the positive animals of step 3 and isolation of M. bovis from lesions. All the positive animals of step 3 (µ i Q(i,3) = 1) are slaughtered (m = 1) and bacterial culture is performed from observed lesions. Sensitivity (Se): sequential combination of a visual inspecti [file pone.0108584.s002.tif]
