# Supplementary material for: Modeling Tuberculosis Dynamics, Detection and Control in Cattle Herds
Source: PLoS One. 2014 Sep 25;9(9):e108584. doi: 10.1371/journal.pone.0108584 (PMC4177924; doi:10.1371/journal.pone.0108584)
Supplement: Appendix S1 — Model equations. (DOCX) [file pone.0108584.s003.docx]

**Appendix S1. Model equations**

The within-herd bTB infection dynamics results from the interaction between three sequential processes: the demographic process (ageing and renewal of animals), the infectious process (transmission of infection and evolution of infected animals) and the process of detection and control.

***Demographic process***

The ageing process (change in age class each January, when *m* = 1) and renewal of animals is represented by the following equations:

$$X_{t}^{(a)}\left( 0,S,0 \right)=\left( 1-1_{m=1} \right)\left( X_{t}\left( 0,S,0 \right)-R_{t}\left( 0,S,0 \right) \right)+\sum_{(i,j,k)\in A\times H\times D} R_{t}\left( i,j,k \right)+M_{t}(i,j,k)$$

$X_{t}^{(a)}\left( 0,j,k \right)=\left( 1-1_{m=1} \right)\left( X_{t}\left( 0,j,k \right)-R_{t}\left( 0,j,k \right) \right)$ if $\left( j,k \right)\neq(S,0)$

$X_{t}^{(a)}\left( i,j,k \right)=\left( 1-1_{m=1} \right)\left( X_{t}\left( i,j,k \right)-R_{t}\left( i,j,k \right) \right)+1_{m=1}(X_{t}\left( i-1,j,k \right)-R_{t}\left( i-1),j,k \right)$ if $i>0$

where:

- $X_{t}^{\left( a \right)}(i,j,k)$ represents the number of animals in age class *i* ($i\in A$), health state *j* ($j\in H$), which have the status *k* ($k\in D$) with respect to the control program, after implementation of the demographic process
- $R_{t}\left( i,j,k \right)$ is the number of animals culled at time step *t*, drawn by the culling rate $\mu_{m}\left( i \right)$specific to *m,* the month of the year and to *i*, the age class of animals:

$$R_{t}\left( i,j,k \right)\sim Binom(X_{t}\left( i,j,k \right), \mu_{m}(i))$$

- $M_{t}(i,j,k)$ is the number of animals slaughtered at time step *t* under the control program
- $1_{condition}$ is the indicator function that equals 1 if the specified condition is true and 0 otherwise.

***Infectious process***

The process of within-herd infection transmission is represented by the following equations:

$X_{t}^{(b)}\left( i,S,k \right)=X_{t}^{(a)}\left( i,S,k \right)-n_{t}^{SE}(i,k)$

$X_{t}^{(b)}\left( i,E,k \right)=X_{t}^{(a)}\left( i,E,k \right)+n_{t}^{SE}(i,k)-n_{t}^{EI}(i,k)$

$X_{t}^{(b)}\left( i,I,k \right)=X_{t}^{(a)}\left( i,I,k \right)+n_{t}^{EI}(i,k)$

Where:

- $X_{t}^{\left( b \right)}(i,j,k)$ represents the number of animals in age class *i* ($i\in A$), health state *j* ($j\in H$), which have the status *k* ($k\in D$) with respect to the control program, after initiation of the infectious process
- $n_{t}^{EI}(i,k)$ is the number of animals that finish the latency period (state *E*) and pass into the state *I*. This number is drawn as a function of the duration of the latency period:

$n_{t}^{EI}(i,k)\sim Binom(X_{t}^{\left( a \right)}\left( i,E,k \right),1-\exp\left( -\alpha\right))$, where $1/\alpha$ is the duration of the latency period in months

- $n_{t}^{SE}(i,k)$ is the number of animals that are infected at time step *t* (and make the transition from the health state *S* to health state *E*). This number is calculated as a function of the force of infection, $\lambda_{t}\left( i \right)$ , which applies to the batch of animals of age class *i*:

$$n_{t}^{SE}(i,k)\sim Binom(X_{t}^{\left( a \right)}\left( i,S,k \right),1-\exp(-\lambda_{t}\left( i \right))$$

- The force of infection is calculated assuming a frequency-dependent transmission of infection:

$$\lambda_{t}\left( i \right)=\beta_{m}\frac{\sum_{(i',k)\in L(i)\times D} X_{t}^{(a)}(i^{'},I,k)}{\sum_{(i',j,k)\in L(i)\times H\times D} X_{t}^{(a)}(i^{'},j,k)}$$

Maintenance conditions of the animals change with the seasons, and these conditions influence the efficiency of the transmission of infection. The parameter of transmission of infection,$\beta_{m}$is assumed to vary according to the month *m*.

***Screening and control process***

The transition from one stage of the control program to another according to the results of slaughterhouse surveillance and planned monitoring is described by the following equations:

$$y_{t+1}=\Phi\left( y_{t}, n_{t}^{evt}, R_{t}, n_{t}^{pos}, n_{t}^{test} \right)$$

$$z_{t+1}=t+\Delta\left( y_{t}, n_{t}^{evt}, R_{t}, n_{t}^{pos}, n_{t}^{test} \right)$$

Where:

- $\Phi$ is the transition function of the control program; $\Phi(y,a,b,c,d)$ returns the number of the next stage of the control program (*y’*) when in step y, *a* animals with lesions are observed among *b* animals culled and *c* animals test positive among *d* animals tested. Similarly, $\Delta(y,a,b,c,d)$ returns the time (in months) at which the tests scheduled in the next stage of the control program should be conducted.
- $n_{t}^{evt}(i,k)$ is the number of culled animals for routine slaughter with bTB-like lesions detected at slaughterhouse. This number depends on the sensitivity of slaughterhouse surveillance (joint sensitivity of the visual inspection of carcasses and of the diagnostic tests performed on lesions); the specificity is assumed to be perfect: $n_{t}^{evt}(i,k) \sim Binom(R_{t}\left( i,I,k \right), {Se}_{ev})$

The implementation of the current step of the control program and its impact on the herd are described by the following equations:

$$X_{t+1}\left( i,j,y_{t} \right)=(1-m_{y_{t}})\sum_{(i,j,k)\in A\times H\times D} n_{t}^{pos}(i,j,k)$$

$X_{t+1}\left( i,j,k \right)=X_{t}^{(b)}\left( i,j,k \right)-\left( 1-m_{y_{t}} \right)n_{t}^{pos}\left( i,j,k \right)-M_{t}(i,j,k)$ for $k\neq y_{t}$

$$X_{t+1}\left( i,j,0 \right)=X_{t}^{(b)}\left( i,j,0 \right)-\left( 1-m_{y_{t}} \right)n_{t}^{pos}\left( i,j,0 \right)-M_{t}(i,j,0)+\sum_{k\geq y_{t}} X_{t}^{(b)}\left( i,j,k \right)$$

$X_{t+1}\left( i,j,k \right)=0$ for $k\geq y_{t}$

Where:

- $n_{t}^{pos}(i,j,k)$ is the number of animals testing positive, selected randomly according to the sensitivity and specificity of the tests:

$$n_{t}^{pos}(i,j,k) \sim Binom\left( n_{t}^{test}\left( i,S,k \right), 1-{Sp}_{y_{t}} \right)+\sum_{j'\in\left\{ E,I \right\}} Binom\left( n_{t}^{test}\left( i,j',k \right), {Se}_{y_{t}} \right)$$

- $n_{t}^{test}(i,j,k)$ is the number of animals to be tested for the current step, $y_{t}$, of the control program (tests are performed if the date has been reached, i.e. when: $1_{t=z_{t}}=1$), and the animals are selected according to the sampling plan $Q_{y_{t}}$:

$$n_{t}^{test}\left( i,j,k \right)=1_{t=z_{t}} Binom(X_{t}^{(b)}\left( i,j,k \right), Q_{y_{t}}\left( i,k \right))$$

- $M_{t}\left( i,j,k \right)$ is the number of animals that had to be slaughtered to perform diagnostic tests:$M_{t}\left( i,j,k \right)=m_{y_{t}} n_{t}^{test}(i,j,k)$
