# Supplementary material for: Modeling Tuberculosis Dynamics, Detection and Control in Cattle Herds
Source: PLoS One. 2014 Sep 25;9(9):e108584. doi: 10.1371/journal.pone.0108584 (PMC4177924; doi:10.1371/journal.pone.0108584)
Supplement: Appendix S2 — Choice of summary statistics. (DOCX) [file pone.0108584.s004.docx]

**Appendix S2. Choice of summary statistics**

The choice of the best combination of summary statistics was made in 3 steps.

***Step A:*** simulation of pseudo-observed data:

1. Arbitrary determination of values ​​for the three parameters to be estimated: *α*=1/12 months, *β_inside_* =0.50 and *β_outside_* =0.10.
2. Use of these parameter values for simulating 100 sets of fictitious data, representing the spread of infection within each of 27 modelled herds.
3. Choice of a single dataset among the 100 simulated datasets that was the closest to the median percentage of animals with lesions; this dataset is referred to below as the pseudo-observed dataset (containing data from 27 fictitious herds).

***Step B:*** calculation of parameter posterior distributions using ABC for each of the summary statistics in Table 3 (the first two points are common to all summary statistics).

1. Attribution of the prior distributions (as described in the parameter estimation section) for the parameters to be estimated: (*α*, *β_inside_* and *β_outside_*).
2. Computation of 100,000 simulations with the model.
3. Calculation of summary statistics for the set of pseudo-observed data.
4. Calculation of summary statistics for the 100,000 simulations.
5. Use of the local linear regression algorithm of the ABC method to estimate the posterior distributions of the parameters, with the proportion of retained simulations, p_ε_ =5%.
6. Verification whether parameter values that were fixed in step A are in the 95% credible interval of obtained posterior distributions.

Once steps A and B were completed, we selected the combinations of summary statistics for which parameter values fixed in step A were in the 95%credible interval of obtained posterior distributions.

***Step C:*** To choose the best summary statistics among those selected in step B, we used all the 100 simulated datasets that were obtained in step A from arbitrarily fixed parameter values of *α*, *β_inside_* and *β_outside_*. Here, instead of using a single set of data as a pseudo-observation, we used all the 100 simulations as pseudo-observations. Then, we repeated step B 100 times, using the 100 pseudo-observations for the ABC method, in order to obtain 100 posterior distributions of the parameters to be estimated (*α*, *β_inside_* and *β_outside_*). We retained the combination of summary statistics that maximised the percentage of cases where the 3 values fixed in step A for the parameters *α*, *β_inside_* and *β_outside_* were within the credible interval (95%) of posterior distributions of this parameters.
