# Supplementary material for: Modeling Tuberculosis Dynamics, Detection and Control in Cattle Herds
Source: PLoS One. 2014 Sep 25;9(9):e108584. doi: 10.1371/journal.pone.0108584 (PMC4177924; doi:10.1371/journal.pone.0108584)
Supplement: Appendix S3 — Effective reproductive rate R(t). (DOCX) [file pone.0108584.s005.docx]

**Appendix S3. Effective reproductive rate**

For each simulated herd, we performed 1,000 simulations; and at each time *t*, we calculated the R(t) by the following formulas:

$$R(t)= C(t) \times\delta(t)$$

$$C\left( t \right)= \frac{1}{t} \frac{\sum_{\tau=1}^{t} \sum_{\left( i,j \right)\epsilon AxD} n_{\tau}^{SE}\left( i,k \right)}{\sum_{\tau=1}^{t} \sum_{\left( i,j \right)\epsilon AxD} X_{\tau}^{\left( b \right)}(i,I,k)}$$

In these expressions, *t* is the current time step, $C \left( t \right)$is the average number of secondary cases per infectious case per step of time during the entire simulation period (time between the start of simulation and the current time step *t*).

$$\delta(t)= \frac{\sum_{\tau=1}^{t} \sum_{\left( i,j \right)\epsilon AxD} X_{\tau}^{(b)}\left( i,I,k \right)}{\sum_{\tau=1}^{t} \sum_{\left( i,j \right)\epsilon AxD} n_{\tau}^{(EI)}\left( i,k \right)}$$

In this equation, $\delta(t)$ is the average duration in months spent by an animal in the state *I* during the entire simulation period.
